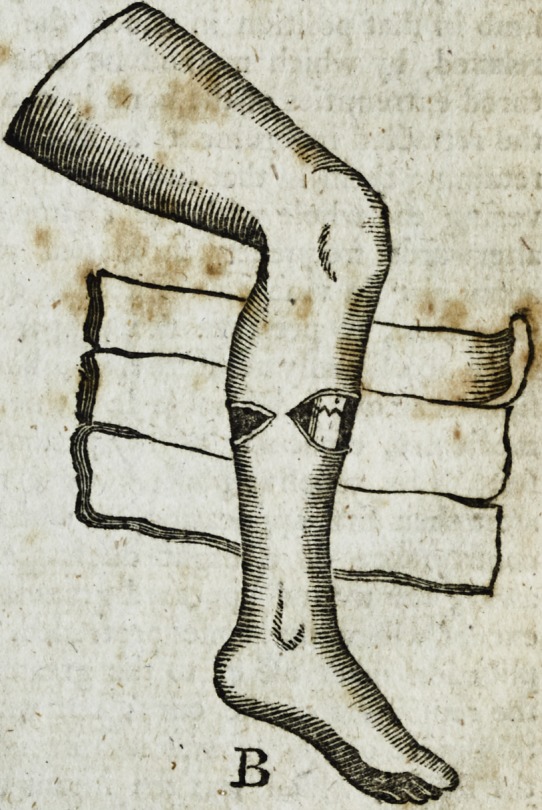# Case of a Compound Fracture of the Leg, with a Considerable Protrusion of the Tibia, Successfully Treated, Principally by the Mode of Healing by the First Intention

**Published:** 1800-06

**Authors:** J. Evans


### *Case of a Compound Fracture of the Leg, with a considerable Protrusion of the Tibia, successfully treated, principally by the Mode of healing by the first Intention.* *By* J. Evans, M. D.

On the 21st of November, 1798, John Vaughan, a boy about eight years of age, being employed in the coal mines in this neighbourhood, at the usual hour of quitting his work; got into a chain fufpended by a rope, in order to be wound up out of the pit, which rope, after paffing over a large iron pulley, was faftened to a wooden machine (commonly called a gin) that was moved round by means of a horfe; which being under the management of a carelefs girl, was fuffered to carry the child with confiderable force againft the pulley, the edges of which form a deep groove for the reception of the rope. The boy being unable to free himfelf, had both the tibia and fibula of his left leg completely fractured, the broken end of the lower part of the tibia protruding upwards for feveral inches, and the integuments with the mufcles were deeply divided in a circular direction round the limb, except a fmall portion of fkin on the infide of the leg, near where the bone pufhed out. Had not the horfe been fortunately ftopped at the moment he was, and the boy extricated from his perilous fituation by a young woman who ran to his affiftance, the limb muft inevitably have been amputated. Being from home at the time the accident happened, Mr. Thomas Dugard, an ingenious young gentleman, a pupil of mine, went immediately to his relief; when, upon his arrival, he found the leg in the fituation before defcribed, without much hæmorrhage.* He directly placed the limb in that pofition in which the mufcles appeared to be moft relaxed, by which method he was enabled to bring the fractured extremities of the bone in contact; over which he brought the retracted integuments as clofe together as he poffibly could, retaining them in that fituation by flips of fticking plafter, covering the whole with the eighteen-tailed bandage, which was afterwards frequently moiftened with a folution of crude fal ammoniac in vinegar. Flexible deal fplints were ufed at the fame time, to preferve the proper form of the limb. Opiates were occafionally adminiftered; but as no fever or much tenfion came on, I did not judge it neceffary to direct any opening medicines, efpecially as my patient's bowels were quite regular. The adhefive plafters were not removed of fixteen days after their firft application, at which time they became loofe in confequence of a flight difcharge. When they were taken away, the wound appeared granulated and united in every part, except where the bone protruded. Dry lint was the only application made ufe of to the greateft part of the wound during the remainder of the cure; but where the tibia was expofed, a thin piece of fponge was laid over it, with a view of abforbing the matter, which was the means of preventing the limb being frequently

* It is well known to men of experience in furgery, that lacerated wounds are feldom attended with profufe hæmorrhage.

frequently diſturbed for the renewal of the bandage. In the courſe of ſix weeks the callus was ſo firm, that the child could bear the leg to be lifted up without making the leaſt complaint; and at the end of two months from the time of the accident, was able to move it in any direction, of his own accord. A portion of the tibia exfoliated as far as it was denuded of its perioſteum; and a piece of the fibula, which had been broken off, made its way through a part of the wound on the under ſide of the leg. At the expiration of four months, the boy returned to his uſual employ with a well-formed limb, equal in length with the other.

REFERENCE to the DRAWING.

A. The appearance of the limb immediately after the accident.

B. The repreſentation of the limb after the ends of the bone were brought in contact.

## Figures and Tables

**Figure f1:**
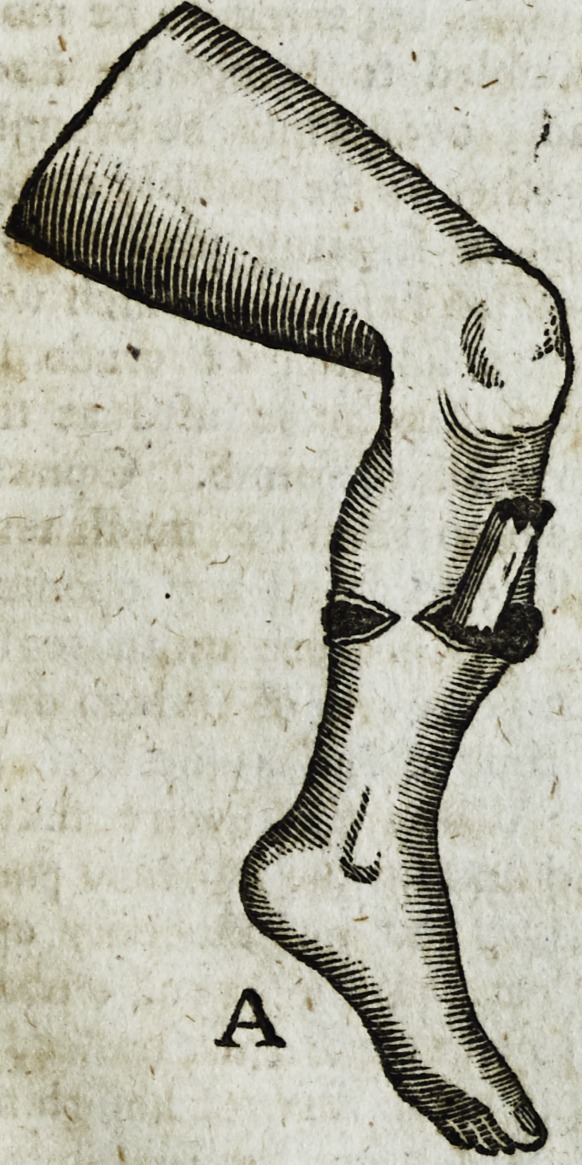


**Figure f2:**